# A Comparison of Dependence across Different Types of Nicotine Containing Products and Coffee

**DOI:** 10.3390/ijerph15081609

**Published:** 2018-07-30

**Authors:** Karl Fagerstrom

**Affiliations:** Fagerstrom Consulting, 18531 Vaxholm, Sweden; karl.fagerstrom@hotmail.com

**Keywords:** dependence, nicotine, tobacco, coffee

## Abstract

Introduction: Few studies have compared the dependence to different tobacco and nicotine products. Even less is known about how it relates to dependence on other common drugs, e.g., caffeine. In this study degree of dependence was compared between snus, cigarettes, nicotine replacement (NR), electronic cigarettes and coffee. Methods: A random sample of Swedish citizens belonging to an internet panel were contacted from September to October 2017. The responders were asked among other related things about their use of snus, NR, traditional cigarette or e-cigarette use and coffee consumption. The indicators of dependence used were: (A) the Heavy Smoking Index, (B) The proportions that used within 30 min after raising in the morning, (C) rating the first use in the morning as the most important and (D) Stating that it would be very hard to give up entirely. Results: Significantly fewer coffee drinkers started use within 30 min of awakening compared with all other products. The first use of the day was found to be more important for snus users compared with other products. On HSI there was no difference between snus and cigarettes. Snus and cigarettes were rated as being more difficult to give up than NR and coffee. Conclusion: Dependence to traditional cigarettes and snus seem to be relatively similar while NR was rated lower and coffee lowest. Since the prevalence of caffeine use in all forms is so much more prevalent than nicotine there might be more persons in the society heavily dependent on caffeine. Implication: Tobacco products are likely more dependence forming than NR products and coffee although there might be more people dependent on caffeine. The addiction to coffee or caffeine is seldom discussed in the society probably because of the little or no harm it causes. Funding: The Snus Commission in Sweden (snuskommissionen) funded the data collection. No funding used for the analysis and writing of manuscript.

## 1. Introduction

Nicotine and tobacco products are associated with dependence [1]. Some studies have found that tobacco products, and particularly cigarettes, are associated with a dependence degree equal to common illicit drugs [2]. As much as tobacco and nicotine products (TNP) can vary in their harmfulness and can be positioned on a continuum of harm there may also be a continuum of dependence. The large majority of studies on dependence on tobacco products has been directed at cigarettes. There are few if any studies comparing the degree of dependence across different nicotine and tobacco products and to other drugs in the same population. The world’s probably most used drug caffeine, which is not a public health problem, is very much ignored when it comes to its dependence potential probably because it is associated with little harm. Officially it is not fully recognised as a drug disorder and needs more research according to American Psychiatric Association. [1]. Little is known how the possible dependence to coffee compares with that of nicotine containing products. In order to get an understanding of this the current study was conducted in a country where different types of nicotine products (NP) are commonly used (cigarettes, snus and nicotine replacement products), to compare the dependence to these products with that to coffee. Thus, there are two questions addressed in this study: how does dependence to coffee compare to dependence to nicotine containing products and does the degree of dependence differ between the nicotine products.

## 2. Methods

### 2.1. Data Collection

A random sample of Swedish citizens belonging to an internet panel were contacted from September to October 2017. The members of the panel were recruited by phone and consists of 67.500 individuals representative of the Swedish population. 8000 panel members were randomly selected and contacted by e-mail. After 2 reminders there were 3001 complete answers giving a response rate of 37.5%. The target population was Swedish citizens and sample size was chosen so that the expected number of smokers, snus users and non-tobacco users, based on prevalence in the population, should be large enough for statistical inference. The sample was confirmed to be representative of the Swedish population according to sex, age, and region.

The questionnaire was cognitively tested to ensure proper understanding. The indicators of dependence used were all taken from the Fagerstrom Test for Cigarette Dependence [3] except for a question on how difficult it would be to stop the tobacco/coffee-related habit. Heavy Smoking Index (HSI), with number of uses/day and time to first use [4] was judged to be relevant for comparing cigarette and snus users because of their similar number of average uses per day but not for coffee or NR use for which frequency of use was lower. Time to first use and importance of the first use were judged to be relevant to ask for all products. The estimation on difficulty to give up each product was made on a 5-point scale. The survey also asked about e-cigarette use but the prevalence of daily use was so low, 0.6%, that it was dropped from all analysis.

Some oversampling was made to increase the sample size of rare events such as daily use of E-cigs and nicotine replacement (NR). The survey was conducted by IPSOS, an opinion poll institute in Stockholm, and supported by the Snuskommissionen (the Snuscommission). www.snuskommissionen.se. A review by a research ethics committee has not been necessary for this type of data collection according to the rules of the Swedish Law on Research Ethics Review. [5].

### 2.2. Statistics

Prevalence was reported as proportions with associated 95% confidence intervals calculated according to the large-sample method for binomial outcomes. Indicators of dependence were compared using two-sample Z test of proportions. HSI scores were compared using Student’s t-test. In pairwise comparisons dual users of tobacco products were excluded. 

Significance level was set at 5%. As multiple tests were used on a single set of data, Bonferroni correction was performed to reduce the risk of obtaining false-positive results (Type I errors). Hence, *p* < 0.003 was considered significant. Statistical analysis was performed using IBM SPSS version 24 (IBM Corp. Released 2016. IBM SPSS Statistics, Version 24.0. Armonk, NY, USA).

## 3. Results

The baseline characteristics of the 3001 participants can be seen in Table 1. For prevalence, which is defined as daily use, it is notable that snus is consumed by more people than cigarettes, 9.9 and 6.8%. NR are consumed by 1.5% of the population while 71% drink coffee daily. The prevalence obtained in this study compares well with the findings of the Swedish Public Health Agency in 2016 that noted a smoking prevalence of 8% for men and 9% for women. The corresponding figures for snus were 18.0% and 4% [6]. A pronounced decline in smoking has been observed the last decades.

In the current study daily average use of cigarettes, snus pouches and cups of coffee was 11.5 (SD = 5.8), 11.7 (SD = 6.6) and 3.6 (SD = 2.3).

There were no differences in HSI score among smokers and snus users (t = 1.7, *p* = 0.12). Use within 30 min was significantly more frequent for snus and cigarettes. For first use coffee was rated by 67% as the most important use and differed significantly from snus and NR. Thirty-five percent of smokers and 33% of snus users stated it would be very hard to give up which was significantly different from the only 18% of coffee drinkers, see Table 2 and Table 3.

Although it seems the dependence to coffee is not as strong as that to cigarettes and snus there might be significantly more people heavily dependent on coffee. In this sample N = 385 would find coffee very hard to give up compared with N = 181 with all TNP together.

Among the participants there was some dual use of products. Thirty subjects used both cigarettes and snus, 8 cigarettes and NR and 4 snus and NR. For coffee the dual use was much higher with 172 using coffee and cigarettes, 256 snus and coffee and 35 NR and coffee.

## 4. Conclusions

First, there was no overall significant difference in degree of dependence between cigarettes and snus. Another Swedish study in youths found that those using snus were as dependent as those smoking [7]. This finding is at odds with data from US that found that cigarette smokers lighted up closer to awakening than smokeless users [8] and that stopping use was easier with smokeless tobacco than with cigarettes [9]. In a study validating a questionnaire assessing dependence to all tobacco products the degree of dependence seemed to be somewhat lower among smokeless tobacco users than cigarette smokers [10]. The conflicting findings could be because the US studies included all sorts of smokeless products which maybe more or less dependence forming or socially acceptable for use, e.g., directly after awakening.

Second, coffee was generally associated with lower dependence than tobacco products except for first use most important that was rated similarly to cigarettes but higher than snus. Significantly fewer used coffee than cigarettes and snus within 30 min.

Third, surprisingly there may be more users of coffee that are heavily dependent in the population than users of TNP because of the much higher numbers of coffee users (2139 than TNP 545). This difference might be smaller in countries with more prevalent smoking.

These results raise the question of how problematic is a strong dependence on coffee when it is associated with very little harm? Addiction or dependence is by itself recognised as a disease [1] and need not involve physical harm but is nevertheless an important indicator in WHO’s International Classification of Diseases and the American Psychiatric Association’s Diagnostic and Statistical Manual. There has not been as much concern about people drinking coffee and maybe becoming heavily dependent as with dependence on TNP. More lately though there has been a discussion on the effects of sodas and energy drinks high in caffeine [11]. In the present study the only caffeine use asked about was drinking coffee. The prevalence of caffeine use would likely have been even higher if daily use of energy drinks, sodas and tea had been included. If a significant proportion of the population being dependent on coffee is not seen as problematic, why is our view so different with nicotine? Probably because traditionally most nicotine has been consumed from burned tobacco and the negative health consequences are well known. But how problematic is the dependence to snus and NR with just a fraction of the dangers from cigarette smoking [12]? The use of NR is most likely confined to former smokers since use of NR without a history of usually heavy smoking is very rare [13].

There are several limitations of this study. The most obvious one may be that the assessment instruments are not well validated for comparing TNP use and not at all validated for coffee use. However, some studies have adopted the FTCD to smokeless tobacco with seemingly meaningful results (e.g., [14]). The relatively small sample size and low prevalence of TNP use resulting in small cells and imprecise estimates is another limitation. Finally dual users were excluded, introducing potential for bias. However, due to the low number of participants using multiple products exclusion of dual users in the pairwise comparisons were deemed feasible and the risk of introducing bias small.

In summary, this cross-sectional study found that cigarettes and snus are about equally dependence forming and higher than that from NR. Coffee was associated with less dependence than all of the other TNP but because of its much higher prevalence there may be as many as or more highly addicted to coffee than to nicotine in society.

## Figures and Tables

**Table 1:** Total number and number of daily users of cigarettes, snus, nicotine replacement (NR) and coffee. Values in parentheses are percentages within each sex/age group.

		Total	Cigarettes	Snus	NR	Coffee
Total		3001	203 (6.8)	298 (9.9)	44 (1.5)	2139 (71.3)
Sex	Male	1520 (51)	83 (5.5)	229 (15.1)	24 (1.6)	1186 (78.0)
	Female	1481 (49)	120 (8.1)	69 (4.7)	20 (1.4)	953 (64.3)
Age	–29	437 (22)	32 (7.3)	28 (6.4)	9 (2.1)	184 (42.1)
	30–39	519 (18)	24 (4.6)	50 (9.6)	3 (0.6)	315 (60.7)
	40–49	473 (17)	29 (6.1)	67 (14.2)	5 (1.1)	318 (67.2)
	50–	1572 (43)	118 (7.5)	153 (9.7)	27 (1.7)	1322 (84.1)

NR: nicotine replacement.

**Table 2:** Prevalence and indicators of dependence. Values in parentheses 95% confidence intervals unless otherwise stated.

	Cigarettes	Snus	NR	Coffee
*n*	203	298	44	2139
Prevalence daily use (%)	6.8 (5.9–7.7)	9.9 (8.9–11.0)	1.5 (1.1–1.9)	71.3 (69.6–72.9)
Heaviness of Smoking Index Score (mean, SD)	2.17 (1.29)	2.31 (1.33)	-	-
First use within 30 min (%)	64.5 (57.8–70.9)	67.8 (62.3–72.9)	56.8 (42.1–70.6)	42.9 (40.8–45.0)
First use the most important of all (%)	64.5 (57.8–70.9)	55.0 (49.4–60.6)	45.5 (31.4–60.1)	67.2 (65.2–69.2)
Would be very hard to give up (%)	35.5 (29.1–42.2)	33.6 (28.4–39.1)	20.5 (10.6–34.0)	18.2 (16.6–19.9)
Absolute numbers finding it very hard to give up	72	100	9	389

SD: standard deviation, NR: nicotine replacement.

**Table 3:** Test of proportions among indicators of dependence. *Z* statistics and associated *p*-values.

First Use within 30 min		*Z*	*p*
Cigarettes	snus	−1.73	0.084
	NR	1.35	0.177
	Coffee	5.92	<0.000 *
snus	NR	1.64	0.101
	Coffee	8.07	<0.000 *
NR	Coffee	1.84	0.066
First use the most important of all			
Cigarettes	snus	1.04	0.298
	NR	1.85	0.064
	Coffee	−0.78	0.435
snus	NR	1.50	0.134
	Coffee	-4.16	<0.000 *
NR	Coffee	−3.04	0.002 *
Would be very hard to give up			
Cigarettes	snus	0.86	0.390
	NR	1.70	0.089
	Coffee	5.92	<0.000 *
snus	NR	1.74	0.082
	Coffee	6.21	<0.000 *
NR	Coffee	0.38	0.704

* *p* < 0.003, NR: nicotine replacement.
